# Microalgae: A Promising Future

**DOI:** 10.3390/microorganisms10081488

**Published:** 2022-07-24

**Authors:** Carmela Caroppo, Patrizia Pagliara

**Affiliations:** 1 Water Research Institute, National Research Council (IRSA-CNR), 74123 Taranto, Italy; 2 Department of Biological and Environmental Sciences and Technologies, University of Salento, 73100 Lecce, Italy

Microalgae are photosynthetic unicellular microorganisms that represent an extremely important component of the aquatic ecosystem productivity, diversity, and functioning [1]. Particularly, phytoplankton, adapted to live in suspension in water masses, provide about half (49%) of the global net primary production in marine and freshwater systems [2]. Moreover, microalgae exhibit a high diversity and include species highly distant from an evolutionary point of view [3]. Despite their small size, which ranges between 0.2 and 200 μm, they can contribute to climate change mitigation through carbon fixation [4,5]. Indeed, biological capture and sequestration of carbon using microalgae have been recognized as one of the world’s most important and effective carbon sequestration methods [6,7]. The promising technique can allow CO_2_ capture and recycle into biomass, which in turn could be useful to produce bioenergy and other value-added products. However, because this becomes efficient, it is important to continue investing widely in the research and development of technology and in the knowledge of the microalgal world. 

In our day, climate change represents a serious problem, also because these changes hardly affect trophic structure and dynamics of aquatic ecosystems as well as phenology, and physiological and life-history traits of organisms.

In the Special Issue “A Glimpse into Future Research on Microalgae Diversity, Ecology and Biotechnology”, studies aimed to improve knowledge on the effects of climate changes on phytoplankton communities’ composition and dynamics in freshwater [8] and brackish [9] ecosystems. Dashkova and co-authors [8], using a eutrophic shallow lake mesocosm as a model, demonstrated how the dynamics of structural and morphological changes of phytoplankton responded to N availability under different temperature conditions. These results could contribute to forecast climate change effects on the world’s shallow lake ecosystems. Moreover, the use of recent complementary technology to traditional microscopy such as imaging flow cytometry (IFC) has proved useful for the morphological and structural analysis of phytoplankton and as a useful tool for routine monitoring programs of aquatic ecosystems. 

On the other hand, Okhapkin and co-authors [9] carried out their research in an atypical brackish system (the small gypsum karstic Lake Klyuchik, Middle Volga basin), characterized by high values of water mineralization and low temperatures. This unique and complex ecosystem, for its peculiarity, represents an interesting model system for the investigation of phytoplankton diversity, largely unexplored in this kind of environment. The value of this research derives not only from the increase in knowledge of phytoplankton diversity in specific conditions, but also from providing helpful tools to decision makers for the management and protection of environments of high naturalistic value.

Another ecological paper reported in this Special Issue focused on the bioremediation of the microalgal species that are considered “harmful” for their ability to produce toxins that negatively impact human health, and to induce blooms with detrimental effects on aquatic ecosystems, fisheries, aquaculture, and tourism [10]. As control actions are urgently needed to suppress harmful algal blooms, Stabili and co-authors [11] demonstrated the high filter-feeding capacity of two sabellid polychaetes, *Branchiomma luctuosum* and *Sabella spallanzanii,* on a harmful microalga (the dinoflagellate *Amphidinium carterae*). Even if preliminary, these results could represent a sustainable and environmentally friendly method for the restoration of the aquatic ecosystems and a very advantageous tool for the management of the aquaculture plans. 

In the field of bioeconomy, the use of innovative processes is extremely important to produce biomaterials and bioenergy, reducing, in the meantime, the consumption of virgin resources. Microalgae-based wastewater treatment represents a valid contribution to this practice. This activity has recently received attention due to its low energy demand, the robust capacity of microalgae to grow under different environmental conditions, and the possibility to recover and transform wastewater nutrients into highly valuable bioactive compounds [12]. 

Outdoor open systems (i.e., raceway ponds, bubble columns, flat panels) are considered so far as the most viable method of microalgal cultivation on wastewaters [13], even if complex and dynamic microbial consortia are formed inside [14,15]. A contribution to this field derives from a study, included in this Special Issue, which examined and compared the nutrient removal efficiency, biomass productivity, and microbial community structure of two outdoor pilot-scale photobioreactors (bubble column and raceway pond) [13]. Data obtained, including the characterization of bacterial and eukaryotic communities using a metabarcoding approach and quantitative PCR, highlighted a different behavior and a different composition of the microbial communities, which were subjected to variations of the environmental conditions as well as of the reactors’ operational parameters. Knowing the factors that influence the structure and dynamics of the microbial consortia allowed establishing which parameters influenced the performance of the reactors. These data encourage the use of microalgal polycultures for a more stable production of biomass in outdoor cultivation systems.

In recent years, an increased interest aimed at expanding knowledge on microalgae has been recorded as they represent a world still partially unknown, but which has many intriguing application aspects being considered as next-generation resources with the potential to address urgent industrial and agricultural demands. In this framework, we must consider that microalgae use a network of signals to interact with all the other organisms living in their environment. The signals are often secondary metabolites generally not necessary for their daily functioning, but they play an important role in competition, defense, attraction, and signaling. These molecules that include pigments, sterols, and polyunsaturated fatty acids can have antioxidant and anti-inflammatory effects [16]. However, even if they are recognized for having bioactive properties, they are still largely underexplored and underexploited. 

Among microalgae, cyanobacteria are one of the most investigated microorganisms for biotechnological purposes, as three reports of the Special Issue demonstrate [17,18,19].

*Arthrospira platensis*, known as *Spirulina*, is a cyanobacterium with multiple nutritional and therapeutic properties [20]. These algae are in fact rich in proteins (60%–70% by weight), vitamins (4% by weight), essential amino acids, minerals (zinc, selenium, magnesium), essential fatty acids (Linoleni-Co acid), carotenoids, chlorophylls, and phytosterols. For this reason, their production is primarily destined for dietary supplement markets, and they are commercialized in many countries. *Arthrospira* is also a rich and inexpensive source of the pigment like phycocyanin [21,22]. Phycocyanin is a blue-red fluorescent, water-soluble, and non-toxic biliprotein pigment with recognized therapeutic properties, including antioxidant, anti-inflammatory, immune-modulatory, and anti-cancer activities. A combination of different techniques is generally used to achieve the purification of phycocyanin from crude algae extracts. Therefore, an increasingly addressed research towards product improvement has been observed in recent years. As an example, Nisticò and co-authors [17] developed a protocol to obtain a very high phycocyanin yield from *A. maxima* biomass. It represents a sustainable process for the recovery, fractionation, and purification of phycocyanin from a strain of *A. maxima* cultivated in a farm devoted to producing this molecule with food-grade purity. In addition, the authors adopted a combination of ultrafiltration and diafiltration that allowed the removal of about 91.7% of the DNA from the crude extract. This is of particular interest as it leads to an increase in purity degree in the retentate fraction, thus allowing the production of extracts suitable for other uses including therapeutic and biomedical applications. 

Being that *Arthrospira* is a species largely cultivated for animal and human food additives and as a source of phycocyanin, a bioactive antioxidant molecule, it is important to know the effects of herbicides on this species. In this context, Piro and co-authors [18] investigated the effects of glyphosate, a broad-spectrum herbicide at the center of a large debate on potential toxicity. On the subject, there are different positions with different results, and this once again also happens regarding the effects on *Spirulina*. Indeed, the authors [18] evidenced that a deliberate treatment with glyphosate on the selected cultivation of this cyanobacterium negatively affected the biomass and the photosynthetic pigments, and it induced resistance in *A. maxima* survival. These results demonstrated that the resistance of *A. maxima* to glyphosate effects is mediated by a key enzyme until now not identified in other *Spirulina* species.

Investigations on bioactive compounds are a very promising area in full development. The growing interest is due to the wide range of applications including the food industry, agrochemicals, cosmetics, and pharmaceutical products. Although new bioactive molecules from the microbial world continue to be discovered, this however remains a field that is still little explored. With the investigations on two cyanobacterial strains, Pagliara and co-authors [19] intended to contribute to increasing the amount of information on the properties of these microorganisms, expanding the cyanobacteria range from which new compounds with significant bioactivity could be identified. The two strains, belonging to *Cyanobium* and *Synechococcus* genera, have been previously identified after their isolation from a Mediterranean marine sponge [23,24]. Intriguing is the presence in these cyanobacteria of important compound as BMAA, 2,4-DAB and microcystin, here evidenced for the first time in cyanobacteria isolated from a marine sponge. Moreover, the strong cytotoxic activity observed for aqueous and methanolic extracts of these two cyanobacteria laid the foundation to produce bioactive compounds of pharmacological interest.

The editors hope that the data reported in this Special Issue could represent a useful reference for researchers and managers interested in microalgae and their biotechnological applications. 
